# Supplementary material for: Organisational Social Influence on Directed Hierarchical Graphs, from Tyranny to Anarchy
Source: Sci Rep. 2020 Mar 9;10:4388. doi: 10.1038/s41598-020-61196-8 (PMC7062773; doi:10.1038/s41598-020-61196-8)
Supplement: Supplementary file 1 — Supplementary Information. [file 41598_2020_61196_MOESM1_ESM.pdf]

Supplementary Information
Organisational Social Influence on Directed Hierarchical Graphs, from Tyranny to Anarchy

Charlie Pilgrim[1,*], **Weisi Guo**[2,4], **and Samuel Johnson**[3,4]

[1]The University of Warwick, Centre for Doctoral Training in Mathematics for Real-World Systems, Coventry, CV4 7AL, UK
[2]Cranfield University, Centre for Autonomous and Cyberphysical Systems, Cranfield, MK43 0AL, UK
[3]The University of Birmingham, Mathematics Department, B15 2TT, UK
[4]The Alan Turing Institute, London, NW1 2DB, UK
*charlie.pilgrim@warwick.ac.uk

Trophic Levels

Considering the food web network adjacency matrix, A, such that a_{ij} is the amount of biomass that species j predates from species i. The trophic level of a species, s_j, is defined as the weighted mean of the trophic levels of the species that j preys upon, plus one[1].

$$s_j = \frac{\sum_i a_{ij} s_i}{\sum_i a_{ij}} + 1 \tag{1}$$

This can be simplified by considering the weighted biomass transfer along edges, p_{ij}, found by normalising the biomass transfer along edges to each predator species,

$$p_{ij} = \frac{a_{ij}}{\sum_j a_{ij}} \tag{2}$$

So that equation 1 becomes

$$s_j - \sum_i p_{ij} s_i = 1 \tag{3}$$

Equation 3 describes a linear system.

$$\begin{bmatrix} 1 & -p_{21} & -p_{31} & \dots & -p_{n1} \\ -p_{12} & 1 & -p_{32} & \dots & -p_{n2} \\ \dots & \dots & \dots & \dots & \dots \\ -p_{1n} & -p_{2n} & -p_{3n} & \dots & 1 \end{bmatrix} \begin{bmatrix} s_1 \\ s_2 \\ \dots \\ s_n \end{bmatrix} = \begin{bmatrix} 1 \\ 1 \\ \dots \\ 1 \end{bmatrix} \tag{4}$$

This can be written in matrix notation, where P is the matrix with entries p_{ij} and $\mathbf{1}$ is a vector of 1s.

$$\mathbf{s} = ((I-P)^{-1})^T \mathbf{1} \tag{5}$$

This linear system can be solved with a unique solution with the sufficient condition that every node has a path from at least one basal species, where a basal species is defined as a species that does not prey on any other species.

Equation 5 can be arrived at through consideration of the linear system. Alternatively, the trophic level can be defined in terms of how many levels biomass travels through to get to each species[2], by considering the biomass weighted adjacency matrix, P.

$$s_j = 1 + \sum_i p_{i,j} + \sum_i p_{i,j}^{(2)} + .. + \sum_i p_{i,j}^{(N)} \tag{6}$$

Where N is the maximum number of steps in a food web chain that we wish to consider. This can be written as

$$\mathbf{s} = (I + P + P^2 + ..P^N)^T \mathbf{1} \tag{7}$$

Comparing this to equation 5, it is clear that equation 7 is equivalent if

$$(I + P + P^2 + ..P^N) = (I - P)^{-1} \tag{8}$$

Multiplying both sides by $(I - P)$, we see that this is equivalent if $P^N \to 0$ as $N \to \infty$. Each basal species, by definition, has in-degree zero and so that the associated column in P must be zeroes. The matrix elements, p_{ij}, were defined as being normalised along j so that the non-zero columns of P sum to 1. Give the condition that each node has at least one path form a basal node, we can see that this is sufficient for $P^N \to 0$ as $N \to \infty$, and so equations 5 and 7 are equivalent.

Calculation of Cycles in a Graph

If we consider the squared adjacency matrix of a directed graph, A^2. The elements of this adjacency matrix can be written as:

$$a_{ij}^{(2)} = \sum_{k=1}^{S} a_{ik} a_{kj} \tag{9}$$

That is, $a_{ij}^{(2)}$ gives the number of paths of length 2 in the graph from node i to j. Through induction, it can be seen that $a_{ij}^{(n)}$ gives the number of paths of length n in the graph from node i to j. The diagonal entries of A^n, a_{ii}^n, will therefore give the number of paths of length n that begin and end at node i, which is the definition of a cycle of length n. In order to find the total number of cycles of length n_{cycle} in a given graph we can sum the diagonals of the graph's adjacency matrix raised to that power, given by the Trace.

$$\text{cycle count} = Tr(A^{n_{cycle}}) \tag{10}$$

Equation 10 was used to calculate the cycle counts in Figure **??**(a).

Derivation of $< v(t) >$ for Cycles

Pure cycle

We will begin by considering influence dynamics in a pure cycle, with no basal node. In a pure cycle, all nodes have a single parent node, and so when the majority rule algorithm randomly selects a node it takes on the state of its sole parent. A node will switch state if and only if it is in the opposite state of its parent node. Through symmetry considerations, there must be the same number of nodes ready to switch from state 1 to -1 if selected as vice versa. (The number of "fringes" between blocks of 1 and -1 nodes must be the same on a circle). At each timestep, the transition probabilities of $v(t)$ are therefore:

$$P_{pure}(v \to v+1) = \frac{p_f}{2} \tag{11}$$

$$P_{pure}(v \to v-1) = \frac{p_f}{2} \tag{12}$$

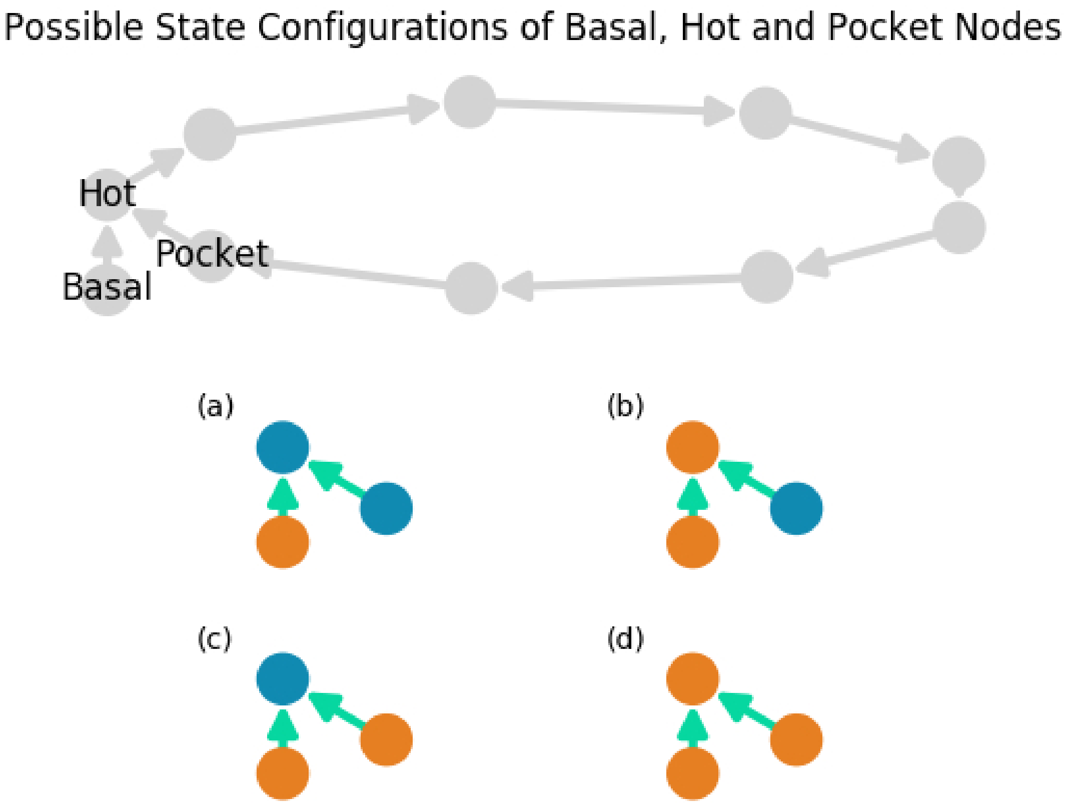

Figure 1. The basal node is always in state -1. The hot and pocket nodes can be in one of 4 configurations of states. Orange nodes here are in state -1, the same as the basal node. Blue nodes are in state 1, the initial state of nodes in the cycle.

$$P_{pure}(v \to v) = 1 - p_f \tag{13}$$

Where p_f is the probability of selecting a child node at a "fringe" where the parent has a different state to the child. There are absorbing states at $v(t) = 0$ and $v(t) = n$, where all nodes are in the same state and there are no fringes. There is some path dependency involved in $p_f(v)$, as certain configurations can only be reached by other configurations. The form of $p_f(v)$ is not necessary for our analysis. From the symmetry between equations 11 and 12 we can see that

$$< v(t) >= v(0) \tag{14}$$

Cycle with a basal node

Now, we add one basal node that influences one of the nodes within the cycle, which we will call the "hot" node. The other important node to consider is the parent of the hot node within the cycle, which we will call the "pocket" node (See Figure 1). We will consider how adding the basal node changes the dynamics from the pure cycle. At each timestep, the only node effected by the basal node is the hot node, and so we can consider the dynamics in the rest of the cycle as proceeding as before.

The basal node is always in state $x_{basal} = -1$. This gives 4 possible configurations for states of the hot and pocket nodes, as shown in Figure 1. For each of these configurations we will write down how the transition probabilities of $v(t)$ differ compared to the pure cycle.

Configuration a. $x_{hot} = 1, x_{pocket} = 1$. Given that the hot node is selected by the algorithm, and that the system begins in configuration a, there is a 50% chance that the basal node will influence the hot node.

$$P_{basal}(v \rightarrow v|hot,a) = \frac{1}{2} \tag{15}$$

$$P_{basal}(v \rightarrow v-1|hot,a) = \frac{1}{2} \tag{16}$$

Comparing this to the pure cycle in configuration a (without a basal node), given that the hot node is selected by the algorithm, there is no chance of any change in $v(t)$

$$P_{pure}(v \rightarrow v|hot,a) = 1 \tag{17}$$

Configuration b. $x_{hot} = -1, x_{pocket} = 1$. Following similar considerations as in Configuration a, we can see that

$$P_{basal}(v \rightarrow v|hot,b) = \frac{1}{2} \tag{18}$$

$$P_{basal}(v \rightarrow v+1|hot,b) = \frac{1}{2} \tag{19}$$

$$P_{pure}(v \rightarrow v+1|hot,b) = 1 \tag{20}$$

Configuration c. $x_{hot} = 1, x_{pocket} = -1$. The pocket and basal node agree, so there is no change from the dynamics of the pure cycle.

$$P_{pure}(v \rightarrow v+1|hot,c) = P_{basal}(v \rightarrow v+1|hot,c) = 1 \tag{21}$$

Configuration d. $x_{hot} = -1, x_{pocket} = -1$. Again, there is no change from the dynamics of the pure cycle.

$$P_{pure}(v \rightarrow v|hot,d) = P_{basal}(v \rightarrow v|hot,d) = 1 \tag{22}$$

Considering equations 15-22, we can adapt the pure cycle transition probability equations 11, 12 and 13 to the basal cycle, in terms of the probability of selecting the "hot" node, $P(hot)$ and the probability of being in configuration m, $P(m)$:

$$P_{basal}(v \rightarrow v+1) = \frac{p_f}{2} - \frac{1}{2}P(hot)P(b) \tag{23}$$

$$P_{basal}(v \rightarrow v-1) = \frac{p_f}{2} + \frac{1}{2}P(hot)P(a) \tag{24}$$

$$P_{basal}(v \rightarrow v) = 1 - p_f + \frac{1}{2}P(hot)P(b) - \frac{1}{2}P(hot)P(a) \tag{25}$$

Assuming large n, we can write the change in the expected value of v as:

$$\frac{d < v >}{dt} \propto P(v \rightarrow v+1) - P(v \rightarrow v-1) \tag{26}$$

Substituting in equations 23 and 24, and adding a constant of proportionality, k,

$$\frac{d<v>}{dt} = -k\left(\frac{1}{2}P(hot)P(a) + \frac{1}{2}P(hot)P(b)\right) \tag{27}$$

$$\frac{d<v>}{dt} = -k\frac{1}{2}P(hot)P(a\cup b) \tag{28}$$

The probability of the algorithm selecting the hot node, $P(hot) = \frac{1}{n}$. Configurations a and b are where the pocket node disagrees with the basal node (Figure 1). The probability that the basal and pocket nodes disagree is equal to $P(a\cup b) = \frac{v}{n}$. Substituting these probabilities in,

$$\frac{d<v>}{dt} = -k\frac{<v>}{2n^2} \tag{29}$$

This can be solved to find that

$$<v(t)>= Ae^{-\frac{kt}{2n^2}} \tag{30}$$

Considering our initial conditions of the basal node being in state -1 while all nodes in the cycle are in state 1:

$$A =< v(0) >= n-1 \tag{31}$$

Equation 30 describes exponential decay. We can replace the constant k by considering the half-life of the decay, in this case how long we expect it to take for half of the nodes in the cycle to be influenced by the basal node through the mechanisms described in equations 23 and 24. The hot node will be selected every n timesteps. In $n-1$ selections of the hot node, we expect, on average, v opportunities for the basal node to change the mechanics from the pure cycle mechanism, of which it will succeed half of the time. The expected half life is therefore $n(n-1)$ timesteps.

$$<v(t)>= (n-1)e^{-\frac{ln2}{n(n-1)}t} \tag{32}$$

References

1. Johnson, S., Domínguez-García, V., Donetti, L. & Muñoz, M. A. Trophic coherence determines food-web stability. *Proc. Natl. Acad. Sci. U.S.A.* **111**, 17923–17928, DOI: 10.1073/pnas.1409077111 (2014).
2. Levine, S. Several measures of trophic structure applicable to complex food webs. *J. Theor. Biol.* **83**, 195–207, DOI: 10.1016/0022-5193(80)90288-X (1980).
